# Galectin signatuares contribute to the heterogeneity of breast cancer and provide new prognostic information and therapeutic targets

**DOI:** 10.18632/oncotarget.7784

**Published:** 2016-02-27

**Authors:** Andrée-Anne Grosset, Marilyne Labrie, Maria Claudia Vladoiu, Einas M Yousef, Louis Gaboury, Yves St-Pierre

**Affiliations:** ^1^ INRS-Institut Armand-Frappier, Laval, Quebec H7V 1B7, Canada; ^2^ IRIC | Université de Montréal, Montreal, Quebec H3T 1J4, Canada

**Keywords:** breast cancer, triple negative, galectins, tissue microarrays, immunohistochemistry

## Abstract

Because of their ability to induce local immunosuppression and to confer cancer cells with resistance to apoptosis, members of the galectin family are emerging as a new class of actionable targets in cancer. Unfortunately, we have yet to obtain a clear picture of the galectin signatures in cancer cells and the surrounding tumor microenvironment. The aim of this study was to provide the first detailed analysis of the galectin signature in molecular subtypes of breast cancer. Expression signatures of galectins were obtained at the mRNA and protein levels. A particular attention was paid to stromal versus epithelial staining and to subcellular compartmentalization. Analysis of the stromal signature showed that gal-1, -3, -9-positive stroma were preferentially found in triple-negative (TN) and HER2 subtypes. In cancer cells, gal-1, −3, -8, and -9 showed a dual expression pattern, being found either in the cytosol or in the cytosol and the nucleus. TN patients with gal-8-positive nuclei had significantly better disease-free survival (DFS), distant-disease-free survival (DDFS), and overall survival (OS). In contrast, high expression of nuclear gal-1 correlated with poor DDFS and OS. TNBC patients who were positive for both nuclear gal-1 and gal-8 had 5-year DFS and DDFS of 100%, suggesting a dominance of the gal-8 phenotype. Overall, the results indicate that specific galectin expression signatures contribute to the phenotypic heterogeneity of aggressive subtypes of breast cancer. Our data also suggest that galectins have clinical utility as indicators of disease progression and therapeutic targets in aggressive molecular subtypes of breast cancer.

## INTRODUCTION

Gene profiling studies have greatly helped at better classifying breast cancer into at least four generally recognized molecular subtypes. Clinically, these molecular subtypes are identified based on the immunohistochemical expression of the estrogen (ER) and progesterone (PR) receptors and of Human Epidermal Growth Factor Receptor 2 (HER2), a member of the epidermal growth factor receptor family. These molecular subtypes include luminal A and B, HER2-positive, and triple negative breast cancer (TNBC; ER-, PR-, and HER2-negative tumor) [1, 2]. Because they lack actionable targets, TNBC and HER2-positive subtypes are thus untreatable with hormone therapies and have a very poor prognosis. The heterogeneity of HER2 and TNBCs at the molecular and cellular levels represents, however, a formidable obstacle to the development of new treatment modalities of these aggressive subtypes [3, 4]. Such challenge is further complicated by the complexity of the tumor microenvironment (TME) which plays a critical role in the disease progression [5].

Carbohydrate-dependent interactions are critical in many physiological processes as well as in pathological abnormalities, most notably in cancer. Outside the cells, these interactions are well known to facilitate intercellular communications, increasing the stability of growth receptors via lattice formation, and modulating the immune response following binding to cell surface receptors [6]. Inside the cells, they modulate signaling cascades, direct trafficking of proteins or contribute to the regulation of gene expression by binding to transcription factors or proteins involved in mRNA splicing [7].

In cancer, such carbohydrate-dependent interactions are mediated in a large part by galectins, an evolutionarily ancient family of soluble proteins that bind N-linked and O-linked beta-galactosides via a conserved Carbohydrate Recognition Domain (CRD). Galectins were first isolated from chick muscle and calf heart and lungs and have since been named in the order of discovery [8, 9]. The 15 members of the family are generally classified according to the number and structure of their CRD. Galectins are therefore divided into tandem, dimeric and chimeric galectins. Dimeric galectins (galectins-1, -2, -5, -7, -10, -11, -13, -14 and -15) have two identical CRD subunits while tandem ones (galectins-4, -6, -8, -9 and -12) have two distinct CRD subunits. Galectin-3 is the only chimeric galectin discovered in mammals thus far. Galectins-5 and -6 are found only in rodents. There is compelling evidence, however, that prototypic galectins might have non-carbohydrate binding partners and functions (reviwed in reference 7). These CRD-independent functions represent a paradigm shift in our understanding of galectin functions.

Our knowledge on the role of galectins in cancer and as biomarkers of disease progression has attracted the interest of many, most notably because these small molecular weight proteins undergo significant changes in their pattern of expression during progression of cancer. Their role in cancer progression, however, is not lacking in subtlety [10]. While cancer progression in accelerated by some galectins, others clearly inhibit tumor growth and/or formation of metastasis. Moreover, there is increasing evidence that galectins function as alarmins [11]. In response to aggression, they are released via a non-classical secretion pathway in the extracellular space where they play a critical role in controlling the immune response. Such complexity in their behavior represents a true challenge when developing galectin inhibitors or use their expression pattern as predictive biomarkers. Moreover, while most studies have examined one galectin at a time, focusing largely on galectin-1 and galectin-3, it is now well established that normal and tumor cells express more than one galectins, and that multiple galectins could be released in the tumor microenvironment (TME) [12, 13]. Defining a galectin signature for specific subtypes is thus critical to identify new therapeutic targets in concert with companion diagnostics and/or molecular signatures to guide therapeutic decisions. In the present work, we have examined the expression of seven galectins in breast cancer tissues by immunohistochemistry and correlated their expression with the different molecular subtypes of breast cancer.

## RESULTS

### *In silico* analysis of galectin mRNA expression in breast cancer tissues

**Figure 1:**
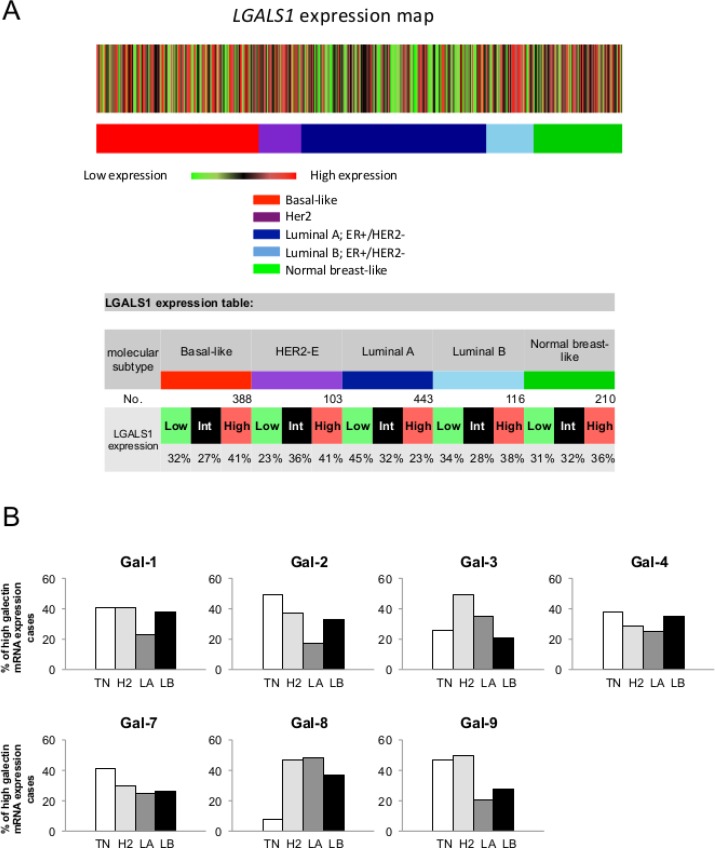
Gene expression map of *lgals1* in PAM50 molecular subtypes of breast cancer using bc-GenExMiner database (**A**) The map shows the percentage of patients with low, medium and high expression of *lgals1* mRNA in each molecular subtype: Basal-like (ER-, HER-2-), HER-2E (HER-2 enriched), Luminal A (ER+, HER-2-, low proliferation) and Luminal B (ER+, HER-2-, high proliferation). The number of patients for each subtypes is shown. (**B**) The distribution for *lgals1, lgals2, lgals3, lgals4, lgals7, lgals8*, and *lgals9* in each molecular subtypes.

We first used the prognostic module of the Breast Cancer Gene-Expression Miner v3.1 (bc-GenExMiner) public database to investigate galectin expression at the mRNA level among breast cancer molecular subtypes. For each subtype, an expression map containing the percentage of patients with low, intermediate, and high gene expression for *gal-1, -2, -3, -4, -7, -8*, and *-9* was retrieved. Gene expression values were being beforehand split in order to form three equal groups so that “high expression” represents the 1/3 of the patients with highest expression of *a gene* and “low expression” is the lower 1/3 of the patients. For example, a representative schematic diagram for *lgals1* shows that *lgals1* expression measured in 1260 samples is at its highest in 41% of patients with basal-like breast and HER2 subtypes of breast cancer (Figure 1A). The diagrams for other members of the galectin family are shown in Supplementary Figure S1. Overall, we found that the relative expression of galectins among the different molecular subtype had a similar distribution, although the percentage of patients with the highest expression of *lgals8*, and to a lesser extent *lgals3*, were lower in patients with a basal-like subtype (Figure 1B).

We next performed an mRNA survival analysis for each galectin using the Breastmark *RNA expression database and algorithm that facilitate* investigation of prognostic markers in the context of disease-free survival (DFS), distant disease-free survival (DDFS) and overall survival (OS) [14]. An initial search was performed for breast cancer as a whole, independently of the lymph node status and across the molecular subtypes classified according the PAM50 molecular classifier [15]. Generation of Kaplan-Meier surviving plots showed no significant differences of DFS between groups of patients with high or low expression level of genes encoding *gal-2, -3, -4, -7, -8*, and *-9* (Supplementary Figure S2). The only notable difference was seen in patients with higher expression of mRNA level of *lgals1*. These patients had a significantly lower (*p* = 0.016) DFS than patients with negative/low levels of *lgals1*, consistent with previous observations that galectin-1 expression correlates with a poor prognosis in breast cancer [16]. High expression of *lgals1* is indeed a poor prognostic factor for both lymph node (LN)-positive and negative breast cancer (Table 1).

**Table 1:** High mRNA expression as a poor prognotic factor for DFS using the pam50 classifier

Gene	Across		LN-pos		LN-neg		Lum A		Lum B		HER2		Basal	
	*n*	*P*	*n*	*P*	*n*	*P*	*n*	*P*	*n*	*P*	*n*	*P*	*n*	*P*
***Lgals1***	2652	**0.016**	744	**0.049**	1183	**0.044**	823	0.410	1013	0.614	286	0.452	424	**0.047**
***Lgasl3***	2601	**0.031**	714	0.546	1161	**0.100**	811	0.663	998	0.313	275	**0.031**	412	0.637
***Lgals7***	455	0.245	173	0.163	173	0.864	153	**0.038**	146	0.200	66	0.965	72	0.220
***Lgals8***	2497	0.726	672	0.218	1105	0.613	783	0.699	950	0.604	273	0.294	407	0.794

**Figure 2:**
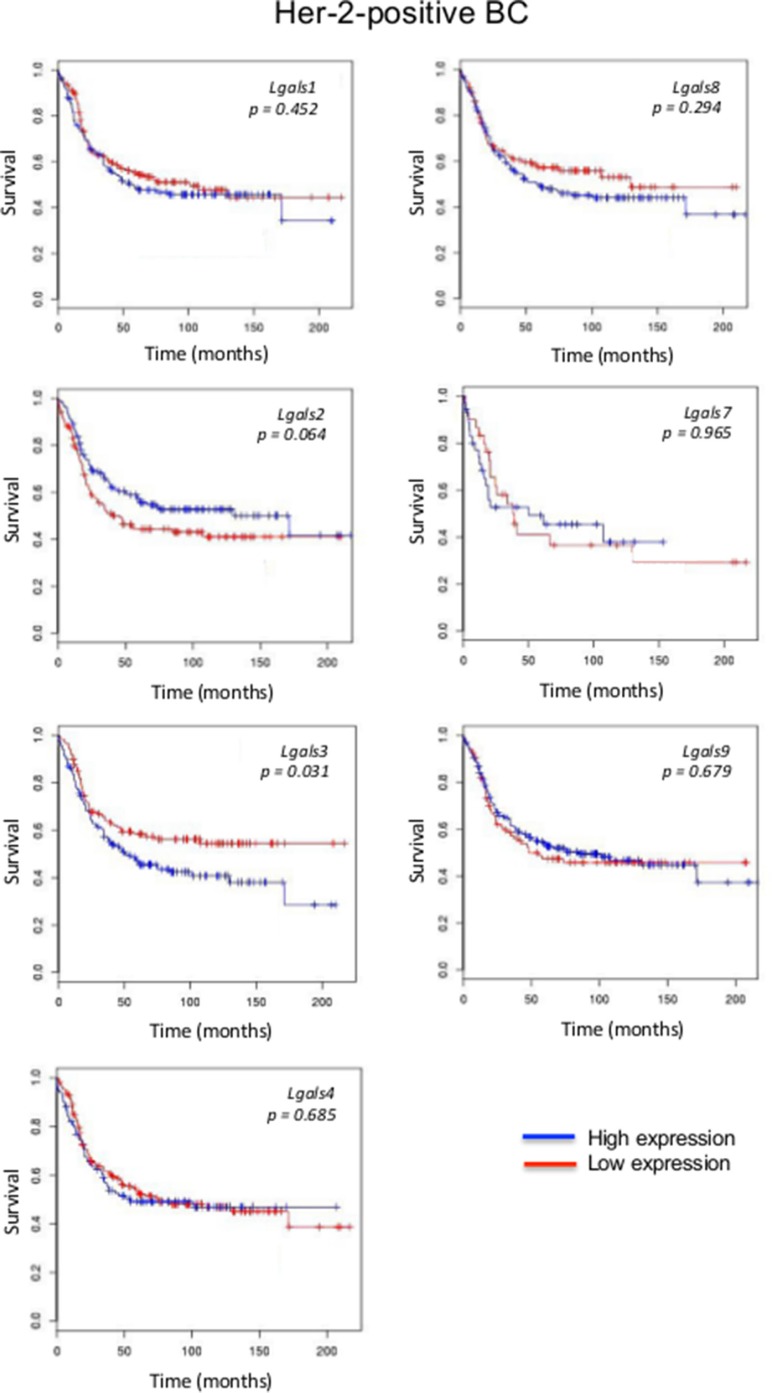
Prognostic role of galectin genes in HER2 breast cancer Kaplan-Meier estimates of overall survival in HER2 patients expressing low or high galectin expression. These figures were generated using BreastMark public database.

**Figure 3:**
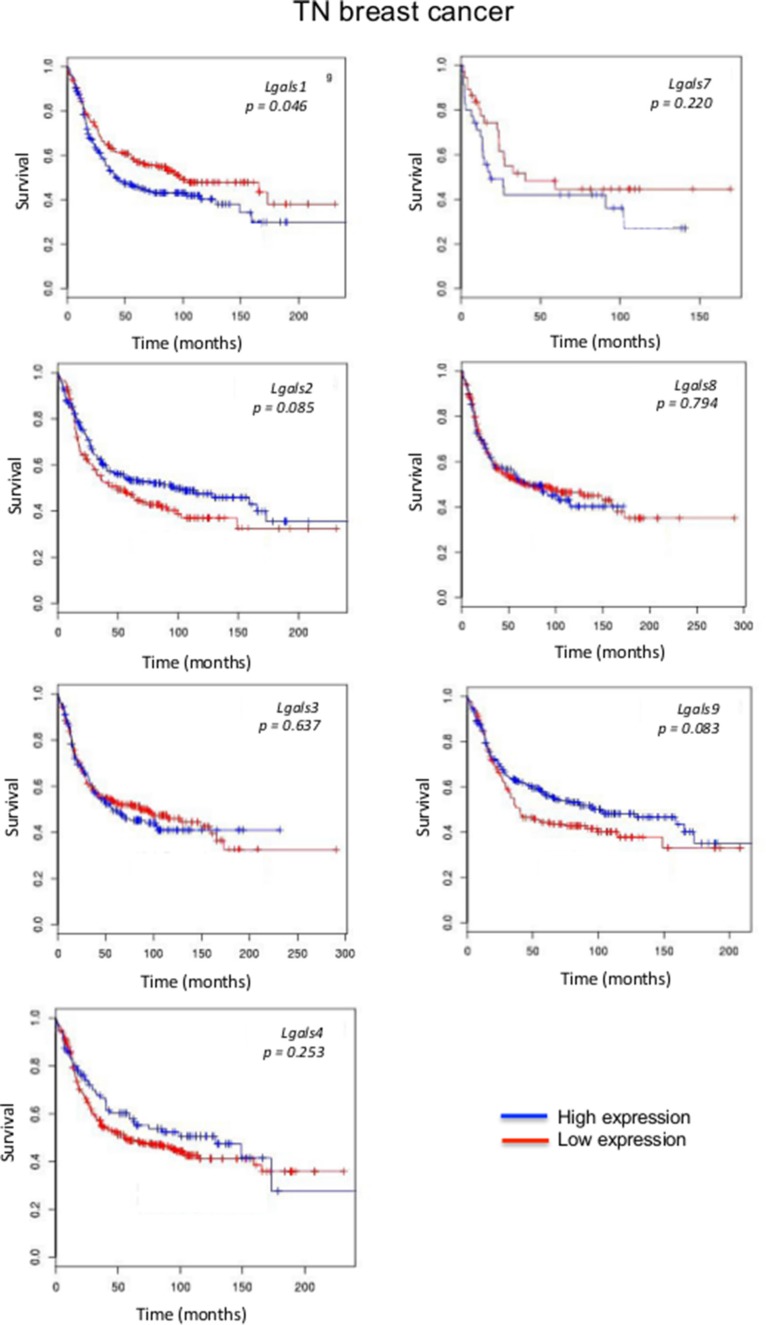
Prognostic role of galectin genes in triple-negative breast cancer Kaplan-Meier estimates of OS in TNBC patients expressing low or high galectin expression. These figures were generated using BreastMark public database.

We next focused on aggressive molecular subtypes for which new prognostics are needed. In the case of HER2 molecular subtype, we found that patients with high *lgals3* gene expression had a significantly (*p* = 0.031) lower OS than patients with lower levels of *lgals3* (Figure 2). In contrast, high *lgals2* expression was associated with a good DFS, although the difference fell just short of the traditional definition of statistical significance (0.064). A similar trend was observed for *lgals2* and *lgals9* in patients with triple-negative breast cancer (Figure 3). High expression levels of *lgals2* (*p* = 0.031), *lgals4* (*p* = 0.061) and *lgals9* (*p* = 0.008) were all good prognostic factors for LN-negative patients (Table 2). *Lgals9* (*p* = 0.004) was also a good prognostic factor for patients with luminal B subtype. Similar results were obtained using the ssp2006 as a classifier [17] (*Data not shown*). Overall, these results indicate that expression of galectins at the mRNA level can be either a good or bad prognostic markers for patients with aggressive subtypes of breast cancer.

**Table 2:** High mRNA expression as a good prognotic factor for DFS using the pam50 classifier

Gene	Across		LN-pos		LN-neg		Lum A		Lum B		HER2		Basal	
	*n*	*P*	*n*	*P*	*n*	*P*	*n*	*P*	*n*	*P*	*n*	*P*	*n*	*P*
***Lgals2***	2952	0.558	719	0.998	1155	**0.031**	807	0.795	987	0.732	275	0.064	419	**0.085**
***Lgals4***	2652	0.281	744	0.537	1183	**0.061**	823	0.449	1013	0.353	286	0.685	424	0.253
***Lgals9***	2652	0.350	744	0.393	1183	**0.008**	823	0.562	1013	**0.004**	286	0.679	424	**0.083**

### Galectin protein signature in normal and cancerous breast tissues

**Figure 4:**
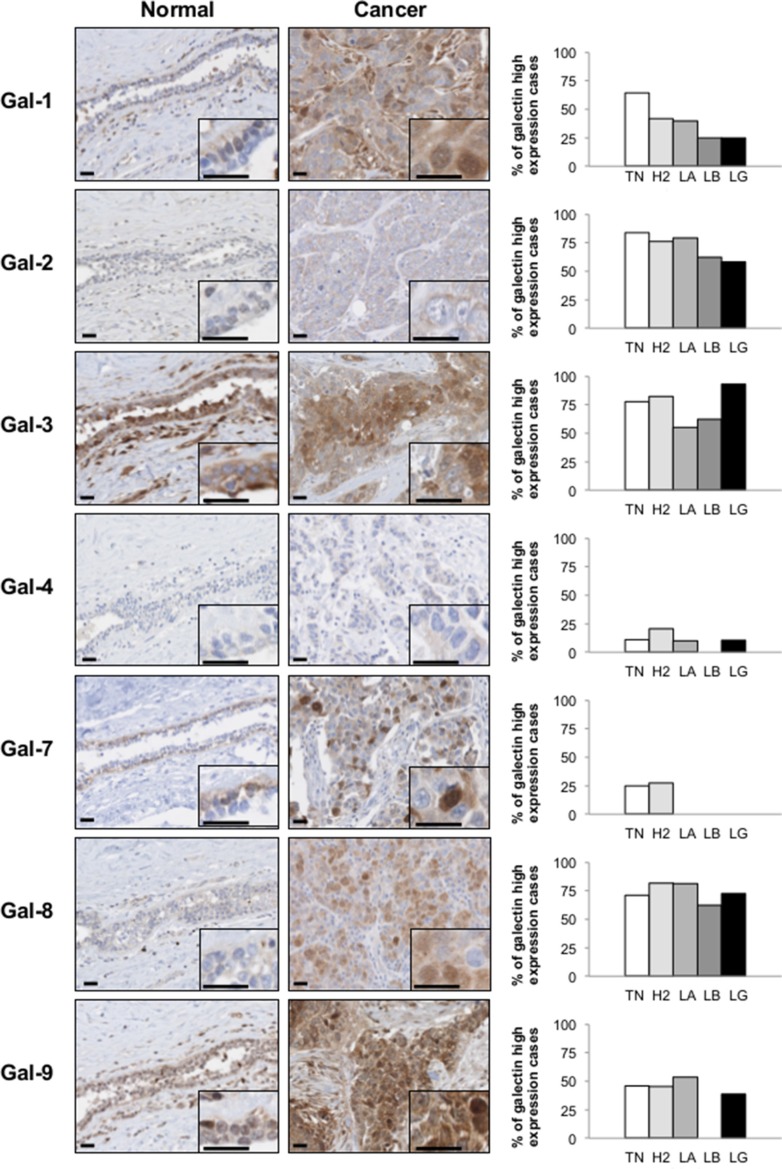
Galectins expression in normal mammary gland and breast cancer tissues *Left panels*, representative images of galectin expression in normal mammary and breast cancer tissues. The histograms on the right represent the percentage of molecular subtypes expressing high levels for each galectin: TN (triple-negative; ER-, PR-, HER-2-), H2 (HER-2 positive; ER-, PR-, HER-2+), LA (Luminal A; ER+, HER-2-), LB (Luminal B; ER+, HER-2+). LG (low grade). All scale bars 25 μm.

**Figure 5:**
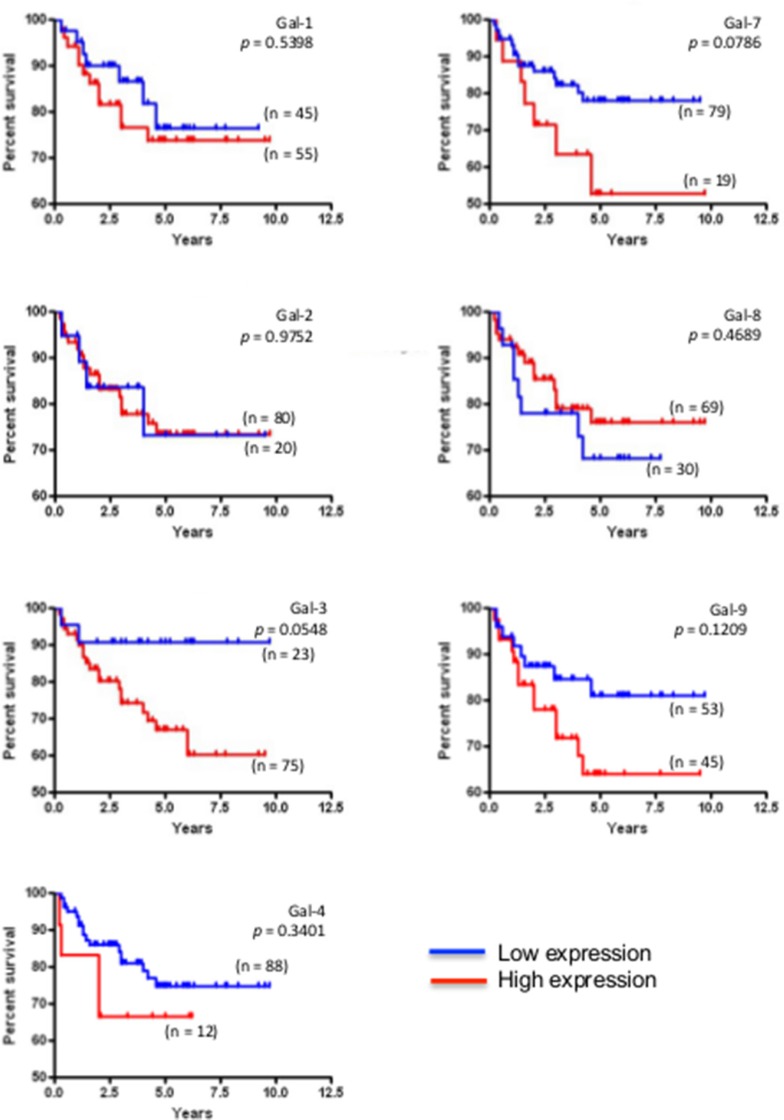
Prognostic potential of galectin in across molecular subtypes of breast cancer Kaplan-Meier estimates of 5-year DFS in breast cancer patients with low or high galectin expression independently of the molecular subtype.

We next studied the expression of galectins at the protein level by immunohistochemistry (IHC) using tissue microarrays (TMAs) constructed from 213 human breast cancer tumor tissues representative of each molecular subtypes of breast cancer defined based on ER/PR/HER2 status and with clinical data. We first validated the specificity of the IHC reactivity of the commercial antibodies using information from the Human Protein Atlas [18, 19] and a review of the literature (Supplementary Table S1). Representative positive IHC staining of tissues for each selected antibody showed a complete agreement with the predictions (Supplementary Figure S3). Once validated, the antibodies were used to examine expression of galectins in normal breast tissues. Our results showed that gal-2 and gal-4 were only weakly expressed in normal breast tissues, with very weak cytoplasmic staining in luminal cells (Figure 4). Also in normal tissues, we found a moderate/high cytoplasmic and/or nuclear staining for all galectins except gal-7, which showed its typical cytoplasmic and nuclear staining in myoepithelial cells [20] (Table 3). Gal-9 staining revealed a cytoplasmic staining in luminal epithelial cells. Some isolated stromal and epithelial cells were also strongly reactive in the nucleus. These patterns of expression in normal breast tissue were, for most galectins, significantly altered in breast cancer tissues. High levels of galectin expression were observed across all molecular subtypes, except for gal-7, which staining was restricted to HER2 and triple-negative breast cancer (TNBCs). Gal-1-positive staining was also preferentially expressed in TNBCs subtype whereas expression of gal-2, -3, -4, -8, and -9 showed an almost equal distribution in all subtypes, although we could not find any gal-9 positive luminal B samples (Table 4). High levels of gal-1 also showed a significant correlation with Ki-67-positive (*p* = 0.048) staining. Generation of Kaplan-Meier surviving plots showed that high expression levels of gal-3 (*p* = 0.0548) and gal-7 (*p* = 0.0786) were associated with a worse DFS across the molecular subtypes although the differences barely missed the commonly acceptable statistical significance of *p* = 0.05 (Figure 5). In the case of gal-3, however, this difference was statistically significant (*n* = 68, *p* = 0.0327) for TNBC (*data not shown*). Moreover, high expression levels of gal-3 in TNBC patients correlated (*p* < 0.05) with recurrence (Supplementary Table S2).

**Table 3:** Galectins distribution, cellular localization and intensity in normal mammary gland

	Breast epithelial cells			Stroma	
	Luminal cells	Myoepithelial cells	Intensity	Localization	Intensity
Gal-1	-	c, n	moderate	e, c, n	high
Gal-2	c	-	weak	e, c	moderate
Gal-3	c	c	high	e, c, n	high
Gal-4	c	-	weak	c	weak
Gal-7	-	c, n	moderate	-	-
Gal-8	c	c	moderate	e, c	moderate
Gal-9	c	c, n	moderate	e, c, n	high

Abbreviations: c, cytoplasmic; n, nuclear; e, extracellular.

**Table 4:** Histoclinical correlations of breast cancers according to galectins expression in cancer cells

	Gal-1 %(n)		Gal-2 %(n)		Gal-3 %(n)		Gal-4 %(n)		Gal-7 %(n)		Gal-8 %(n)		Gal-9 %(n)	
	Neg/Low	High	Neg/Low	High	Neg/Low	High	Neg/Low	High	Neg/Low	High	Neg/Low	High	Neg/Low	High
Molecular subtype	**P = 0.001**								***P* < 0.0001**					
Triple neg	**14 (26)**	**25 (47)**	6 (12)	33 (62)	9 (16)	30 (56)	36 (66)	4 (8)	**29 (54)**	**10 (18)**	12 (21)	29 (52)	21 (39)	18 (33)
HER-2	**10 (18)**	**7 (13)**	4 (8)	14 (26)	3 (6)	15 (28)	15 (27)	4 (7)	**13 (24)**	**5 (9)**	3 (6)	15 (27)	10 (18)	8 (15)
Luminal A	**26 (48)**	**13 (24)**	11 (21)	26 (49)	11 (20)	27 (50)	33 (62)	4 (7)	**39 (71)**	**0 (0)**	8 (15)	29 (52)	20 (37)	18 (33)
Luminal B	**3 (6)**	**1 (2)**	2 (3)	3 (5)	2 (3)	3 (5)	4 (8)	0 (0)	**4 (8)**	**0 (0)**	2 (3)	3 (5)	4 (8)	0 (0)
SBR-EE Grade	***P* = 0.006**		***P* = 0.02**		***P* = 0.017**				***P* = 0.005**					
I (low)	**14 (24)**	**5 (8)**	**8 (13)**	**11 (18)**	**1 (2)**	**16 (28)**	15 (26)	2 (3)	**18 (31)**	**0 (0)**	5 (8)	12 (21)	11 (19)	7 (12)
III (high)	**39 (67)**	**42 (72)**	**17 (29)**	**65 (112)**	**23 (39)**	**60 (102)**	74 (127)	9 (15)	**66 (113)**	**16 (27)**	21 (36)	62 (104)	46 (78)	36 (62)
Age, yr														
≤ 45	9 (16)	11 (20)	5 (9)	15 (27)	4 (8)	15 (27)	16 (30)	4 (7)	16 (30)	4 (7)	3 (5)	16 (29)	12 (22)	8 (15)
> 45	44 (81)	36 (66)	19 (35)	62 (114)	20 (37)	61 (111)	72 (132)	8 (15)	69 (126)	11 (20)	22 (39)	59 (107)	43 (79)	36 (66)
Tumor volume														
≤ 10 cm^3^	31 (52)	26 (44)	14 (23)	42 (72)	12 (20)	44 (75)	47 (80)	8 (14)	49 (84)	7 (12)	13 (21)	45 (75)	31 (52)	25 (43)
> 10 cm3	21 (36)	22 (37)	10 (17)	35 (59)	14 (24)	30 (52)	41 (70)	4 (7)	37 (62)	7 (12)	13 (21)	30 (51)	23 (39)	21 (35)
Lymph node metastasis														
Negative	32 (58)	32 (58)	13 (23)	51 (93)	15 (26)	49 (87)	57 (102)	6 (11)	56 (101)	7 (13)	15 (27)	48 (85)	35 (62)	28 (50)
Positive	20 (35)	16 (28)	11 (19)	25 (46)	9 (16)	28 (50)	31 (56)	6 (11)	29 (52)	7 (13)	9 (15)	28 (49)	20 (35)	17 (31)
ER	***P* = 0.004**								***P* < 0.0001**					
Neg/Low	**27 (50)**	**34 (62)**	12 (22)	51 (94)	12 (22)	50 (92)	55 (101)	8 (15)	**47 (86)**	**15 (27)**	17 (31)	45 (82)	34 (62)	27 (50)
High	**26 (48)**	**13 (24)**	12 (22)	26 (48)	13 (23)	26 (47)	34 (62)	4 (7)	**39 (71)**	**0 (0)**	8 (14)	30 (54)	22 (40)	17 (31)
PR	***P* = 0.034**								***P* = 0.029**					
Neg/Low	**43 (79)**	**43 (79)**	20 (38)	66 (123)	22 (40)	65 (119)	76 (141)	10 (19)	**72 (132)**	**15 (27)**	22(40)	65 (118)	49 (90)	37 (67)
High	**10 (19)**	**4 (7)**	3(6)	10 (19)	3 (5)	11 (20)	12 (22)	2 (3)	**14 (25)**	**0 (0)**	3(5)	10 (18)	7 (12)	8 (14)
HER-2														
Neg/Low	41 (74)	38 (69)	17 (32)	60 (110)	19 (35)	58 (105)	69 (126)	8 (15)	68 (123)	10 (18)	20 (36)	57 (102)	41 (74)	37 (66)
High	13 (24)	8 (15)	6 (11)	17 (31)	5 (9)	18 (33)	19 (35)	4 (7)	18 (32)	5 (9)	5 (9)	18 (32)	14 (26)	8 (15)
Ki-67	***P* = 0.048**								***P* = 0.032**					
Neg/Low	**36 (66)**	**24 (44)**	17 (31)	43 (79)	14 (25)	46 (85)	50 (92)	9 (17)	**54 (99)**	**6 (11)**	16 (29)	43 (76)	35 (63)	25 (46)
High	**18 (32)**	**22 (40)**	7 (12)	34 (63)	11 (20)	29 (53)	38 (70)	3 (5)	**31 (56)**	**9 (16)**	9 (16)	32 (58)	21 (38)	19 (35)

Fisher's exact test and chi-square test.

### Cellular localization of galectins in breast cancer cells

**Figure 6:**
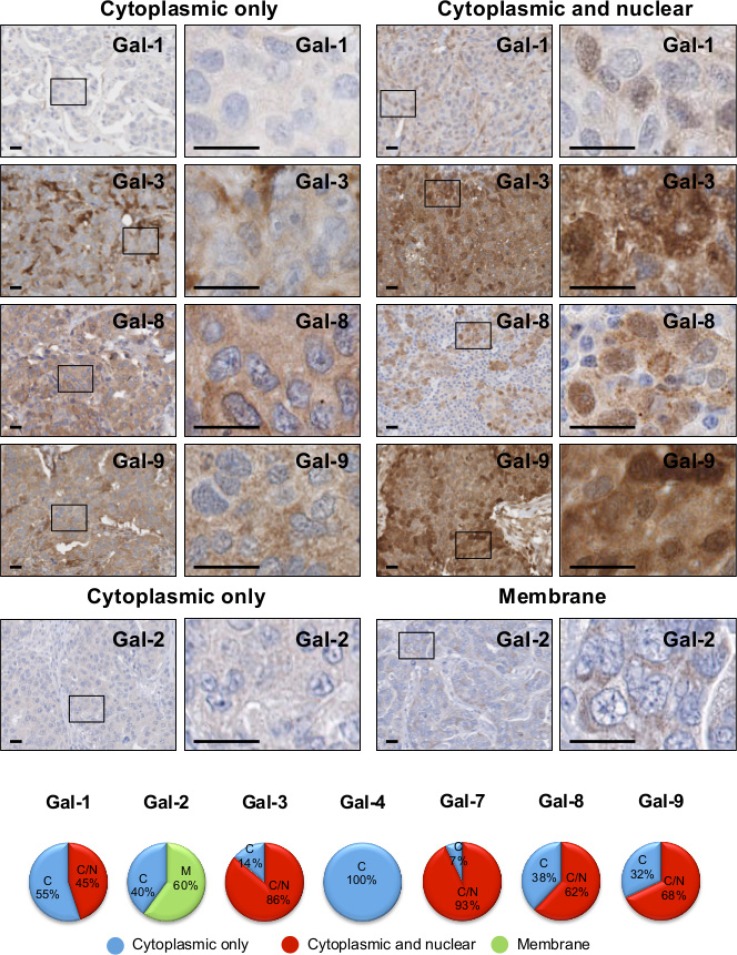
Subcellular localization of galectins in breast cancer tissues Representative IHC stainings of galectin expression in breast cancer tissues. All scale bars, 25 μm. The lower circle graphs show the percentage of galectin-positive staining in the cytosol only (C), at the membrane (M) and in the cytosol and nuclei (C/N).

Because previous studies have shown that galectin functions depends on its subcellular localization [21, 22], we examined the subcellular distribution of galectins in breast cancer cells and its association with tumor progression. We found that gal-1, -3, -8, and -9 have a dual expression patterns in breast cancer cells. Their expression is either restricted to the cytoplasmic compartments or found in the cytoplasm and the nucleus in the same cell (Figure 6). We found no evidence of nuclear localization of gal-2 and gal-4, in contrast to gal-7, which is almost always found in both nuclear and cytoplasmic compartments in the same cell (Figure 4).

### Prognostic values of subcellular galectins

**Figure 7:**
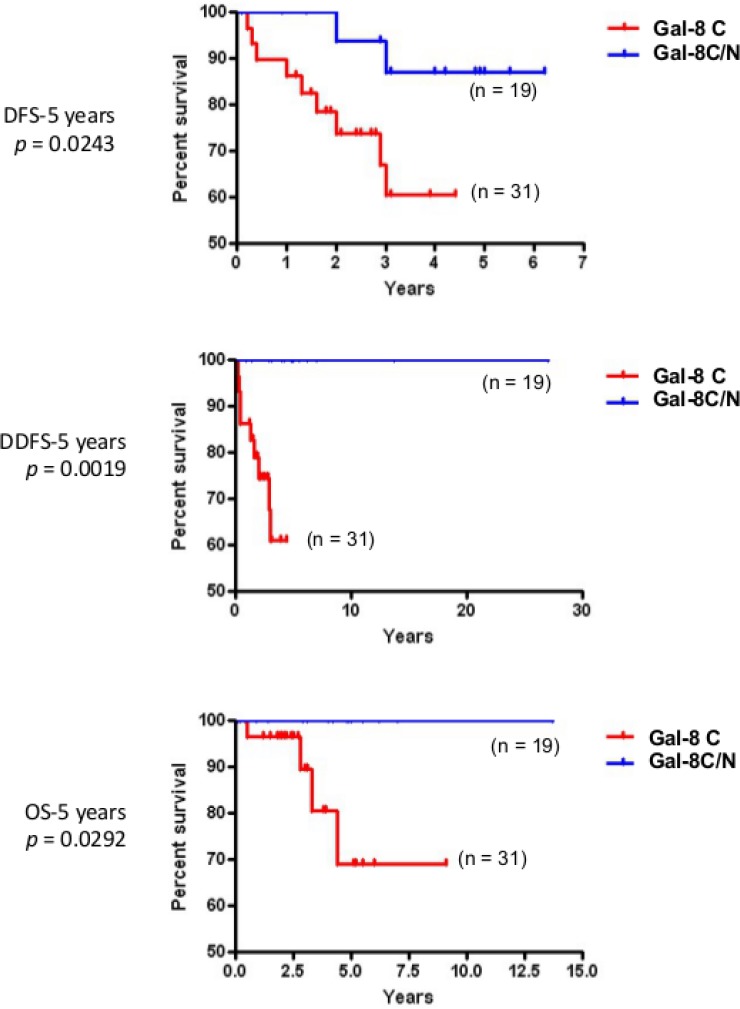
Prognostic potential of nuclear galectin-8 in TNBC Kaplan-Meier estimates of 5-year DFS, DDFS, and OS in TNBC breast cancer patients expressing cytosolic (C) or cytosolic and nuclear (C/N) galectin-8.

**Figure 8:**
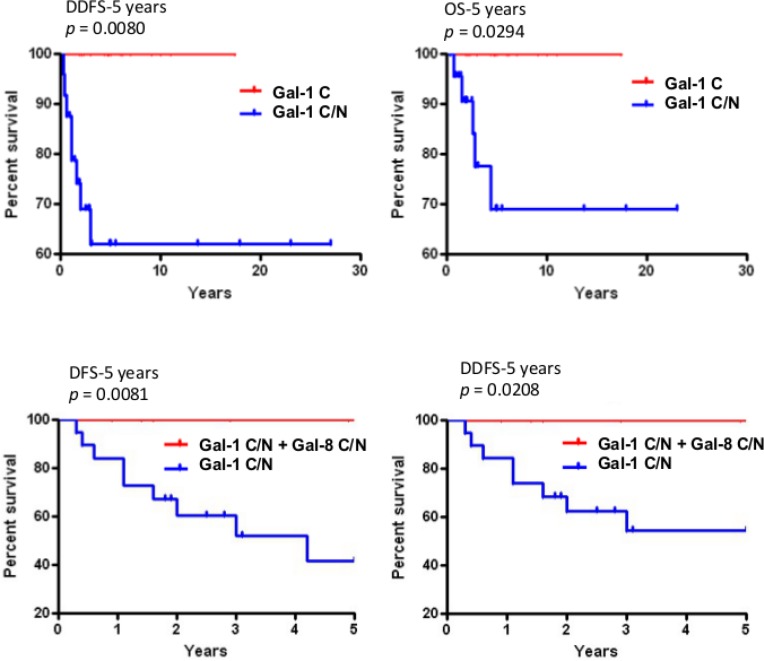
Prognostic potential of nuclear galectin-1 and -8 *Upper histograms*, Kaplan-Meier estimates of 5-year DDFS and OS in TNBC breast cancer patients expressing cytosolic (C) or cytosolic and nuclear (C/N) galectin-1. *Lower histograms*, Kaplan-Meier estimates of 5-year DDFS and DFS in breast cancer patients expressing nuclear galectin-1 and/or galectin-8.

In the case of gal-1 and gal-2, their respective nuclear and membrane localization correlated with TNBCs (Table 5). In contrast, gal-8 nuclear expression was preferentially found in low grade breast tumor and significantly (*p* < 0.0001) less frequent in TNBCs. Membrane-associated gal-2 (*p* = 0.039) also correlated with high expression of Ki-67. In fact, in TNBC, patients with gal-8-positive nuclear staining had significantly better DFS (*p* = 0.0243), DDFS (*p* = 0.0019), and OS (*p* = 0.0292) (Figure 7). Such correlation was also observed independently of the molecular subtype (Supplementary Figure S4). In contrast, high expression of nuclear gal-1 correlated with a worst DDFS (*p* = 0.0080) and OS (*p* = 0.0294) in TNBC (Figure 8). Interestingly, patients who were positive for both nuclear gal-1 and nuclear gal-8 had an actual 5-year DFS and DDFS of 100%.

**Table 5:** Histoclinical correlations of breast cancers according to galectins localization in cancer cells

Characteristics	Gal-1 % (n)		Gal-2 % (n)		Gal-3 % (n)		Gal-8 % (n)		Gal-9 % (n)	
	C	C/N	C	M	C	C/N	C	C/N	C	C/N
Age, yr										
≤ 45	14 (12)	9 (8)	9 (13)	10 (14)	3 (4)	17 (23)	10 (14)	11 (15)	5 (4)	14 (11)
> 45	41 (35)	36 (31)	31 (43)	50 (71)	11 (15)	69 (96)	27 (37)	52 (70)	27 (22)	54 (44)
Tumor volume							***P* = 0.002**			
≤ 10 cm^3^	33 (27)	21 (17)	24 (32)	31 (40)	8 (10)	51 (65)	**16 (20)**	**44 (55)**	14 (11)	41 (32)
> 10 cm^3^	24 (19)	22 (18)	14 (19)	31(40)	7 (9)	34 (43)	**22 (28)**	**18 (23)**	17 (13)	28 (22)
Lymph node metastasis					***P* = 0.043**					
Negative	37 (32)	30 (26)	25 (34)	42 (59)	**6 (8)**	**58 (79)**	22 (29)	42 (56)	21 (17)	41 (33)
Positive	18 (15)	15 (13)	16 (22)	17 (24)	**8 (11)**	**28 (39)**	16 (22)	20 (27)	11 (9)	27 (22)
SBR-EE Grade			***P* < 0.0001**				***P* = 0.024**			
I (low)	6 (5)	4 (3)	**12 (15)**	**2 (3)**	1 (1)	21 (27)	**2 (3)**	**15 (18)**	1 (1)	15 (11)
III (high)	46 (37)	44 (35)	**28 (36)**	**58 (76)**	14 (18)	64 (84)	**35 (44)**	**48 (60)**	30 (22)	54 (40)
Molecular subtypes	***P* = 0.019**		***P* < 0.0001**				***P* < 0.0001**			
Triple negative	**22 (19)**	**33 (28)**	**7 (10)**	**37 (52)**	7 (10)	33 (46)	**24 (33)**	**14 (19)**	17 (14)	23 (19)
HER-2 positive	**9 (8)**	**6 (5)**	**11 (15)**	**8 (11)**	4 (5)	16 (23)	**6 (8)**	**14 (19)**	6 (5)	12 (10)
Luminal A	**22 (19)**	**6 (5)**	**20 (29)**	**14 (20)**	3 (4)	33 (46)	**7 (10)**	**31 (42)**	9 (7)	32 (26)
Luminal B	**1 (1)**	**1 (1)**	**2 (3)**	**1 (2)**	0 (0)	4 (5)	**0 (0)**	**4 (5)**	0 (0)	0 (0)
ER expression	***P* = 0.007**		***P* = 0.007**				***P* = 0.0003**			
Neg/Low	**33 (28)**	**40 (34)**	**21 (30)**	**45 (64)**	11 (15)	55 (77)	**30 (41)**	**30 (41)**	23 (19)	38 (31)
High	**22 (19)**	**6 (5)**	**19 (27)**	**15 (21)**	3 (4)	31 (43)	**7 (10)**	**33 (44)**	9 (7)	30 (24)
PR expression			***P* = 0.042**							
Neg/Low	50 (43)	42 (36)	**32 (45)**	**55 (78)**	12 (17)	73 (102)	35 (47)	52 (71)	28 (23)	54 (44)
High	5 (4)	3 (3)	**8 (12)**	**5 (7)**	1 (2)	13 (18)	3 (4)	10 (14)	4 (3)	14 (11)
HER-2 expression			***P* = 0.023**							
Neg/Low	43 (36)	39 (33)	**27 (38)**	**51 (72)**	10 (14)	66 (91)	31 (42)	45 (60)	26 (21)	56 (45)
High	11 (9)	7 (6)	**13 (18)**	**9 (13)**	4 (5)	20 (28)	6 (8)	18 (24)	6 (5)	12 (10)
EGFR expression	***P* = 0.008**						***P* = 0.044**			
Neg/Low	**49 (42)**	**28 (24)**	35 (49)	46 (65)	12 (16)	66 (90)	**26 (35)**	**54 (72)**	26 (21)	53 (42)
High	**6 (5)**	**17 (14)**	6 (8)	13 (19)	2 (3)	20 (28)	**11 (15)**	**9 (12)**	6 (5)	15 (12)
Ki-67 expression			***P* = 0.039**				***P* = 0.002**			
Neg/Low	33 (28)	19 (16)	**27 (38)**	**29 (41)**	8 (11)	54 (74)	**15 (20)**	**42 (56)**	16 (13)	41 (33)
High	21 (18)	26 (22)	**13 (19)**	**31 (44)**	6 (8)	33 (45)	**23 (31)**	**20 (27)**	16 (13)	27 (22)

Fisher's exact test and chi-square test.

### Stromal galectin expression in stromal cells of the tumor microenvironment

**Figure 9:**
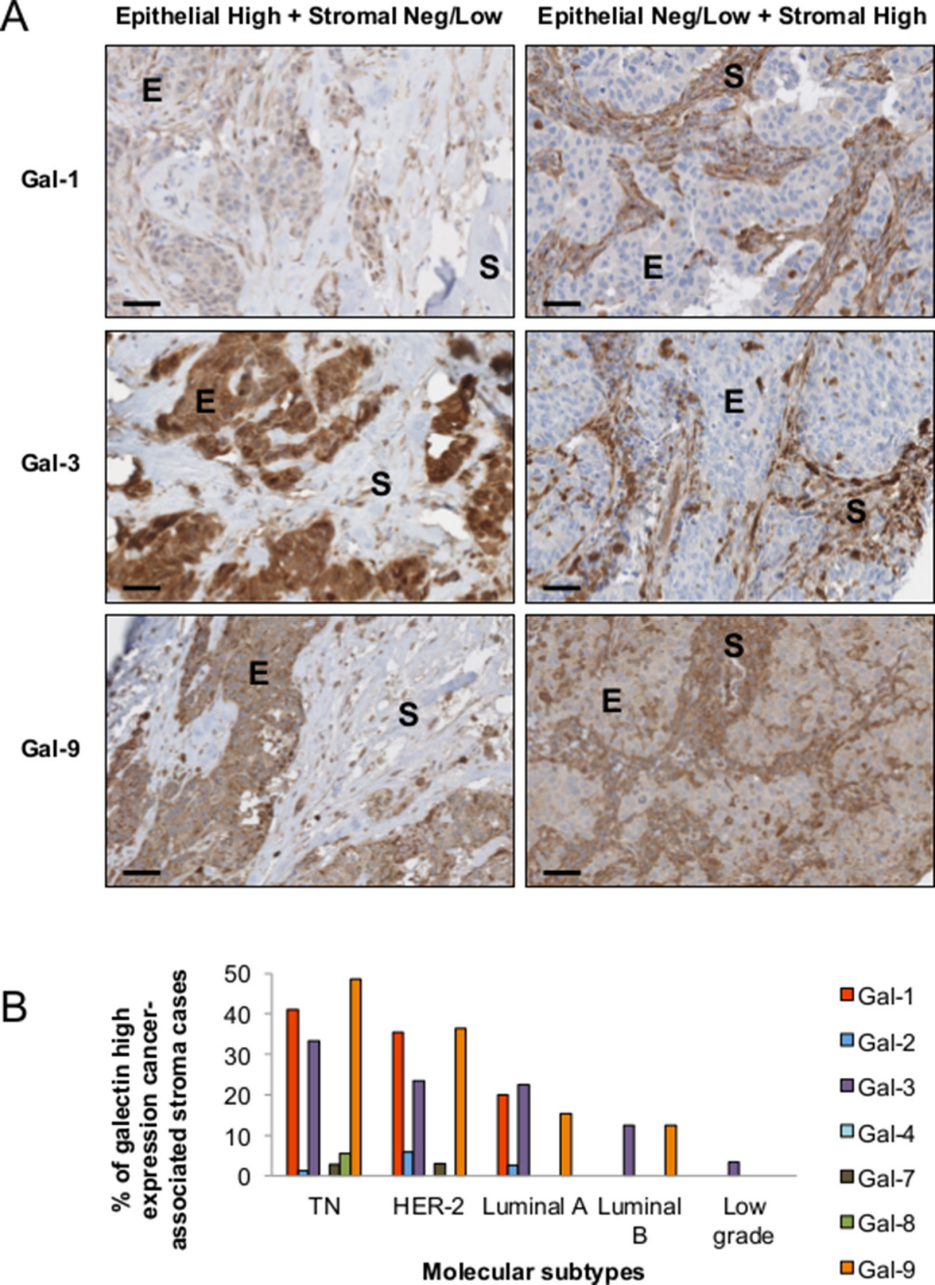
Stromal expression of galectins (**A**) Representative IHC staining of galectin-1, -3, and -9 expression in epithelial (E) and stromal (S) cells in breast cancer tissues. All scale bars, 50 μm. (**B**) Bar histograms showing the percentage of cases with high levels of expression of stromal galectin-1, -3, and 9 in molecular subtypes. (**C**) Correlation between galectin-1, -3, and -9 expression in breast cancer tissues measured across the molecular subtypes.

**Figure 10:**
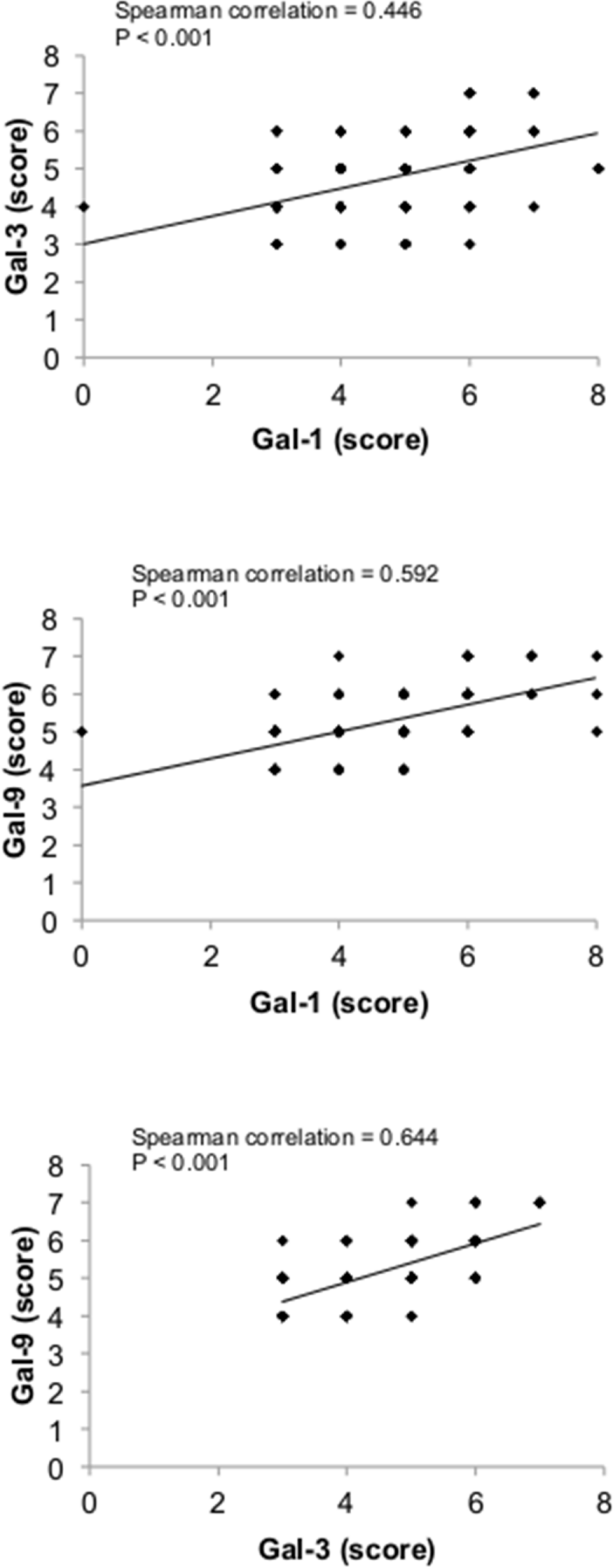
Correlation between galectin-1, -3, and -9 expression in breast cancer tissues Correlations were measured across the molecular subtypes.

There is increasing evidence that the tumor microenvironment is a key contributor to tumor progression [5]. We have thus examined whether galectins are expressed in stromal cells of the tumor microenvironment. We found that gal-1, -3, and -9 are commonly found in cells surrounding the tumor, while expression of other galectins were mostly associated with epithelial cancer cells (Figure 9A). In many cases, staining for gal-1, -3, or -9 were found in both epithelial and stromal cells (approximately 50% in the case of gal-1 and gal-9 and 75% in the case of gal-3). In other cases, we could clearly distinguish two clear patterns of staining with these galectins, i.e. that gal-1, -3, and -9 expression were either strictly found in epithelial tissues or in stromal cells. Gal-1, -3, -9-positive stroma were preferentially found in tumours from TN and HER2 patients (Figure 9B) and correlated with EGFR-positive, Ki67-positive, and mutated p53 (Table 6). Overall, we found that 22 patients were positive for all three galectins (Table 7). All were high grade tumors or were classified as TNBC/HER2 molecular subtypes. We also found a statistically significant positive correlation between expressions of these galectins (Figure 10).

**Table 6:** Histoclinical correlations of breast cancers according to galectins expression in cancer-associated stroma

Characteristics	Gal-1 % (n)		Gal-3 % (n)		Gal-9 % (n)	
	Neg/Low	High	Neg/Low	High	Neg/Low	High
Age, yr						
≤ 45	16 (30)	3 (6)	15 (28)	4 (7)	15 (27)	6 (10)
> 45	57 (104)	24 (43)	61 (112)	20 (36)	54 (98)	26 (47)
Tumor volume						
≤ 10 cm^3^	43 (72)	14 (24)	42 (71)	14 (24)	39 (66)	17 (29)
> 10 cm^3^	29 (49)	14 (24)	35 (59)	10 (17)	29 (49)	15 (25)
Lymph node metastasis						
Negative	48 (85)	17 (31)	48 (85)	16 (28)	42 (74)	21 (38)
Positive	25 (45)	10 (18)	29 (51)	8 (15)	26 (47)	11 (19)
SBR-EE Grade	***P* < 0.0001**		***P* = 0.003**		***P* < 0.0001**	
I (low)	**19 (32)**	**0 (0)**	**17 (29)**	**1 (1)**	**18 (30)**	**1 (1)**
III (high)	**54 (92)**	**28 (47)**	**60 (102)**	**23 (39)**	**51 (87)**	**31 (53)**
Molecular subtypes	***P* = 0.0001**		*P* = 0.0516	***P* < 0.0001**	
Triple negative	**23 (43)**	**16 (30)**	26 (48)	13 (24)	**19 (35)**	**20 (37)**
HER-2 positive	**11 (20)**	**6 (11)**	14 (26)	4 (8)	**11 (21)**	**7 (12)**
Luminal A	**35 (64)**	**4 (8)**	33 (60)	5 (10)	**34 (63)**	**4 (7)**
Luminal B	**4 (8)**	**0 (0)**	4 (7)	1 (1)	**4 (7)**	**1 (1)**
ER expression	***P* < 0.0001**		***P* = 0.031**		***P* < 0.0001**	
Neg/Low	**38 (70)**	**23 (42)**	**44 (81)**	**18 (33)**	**34 (63)**	**27(49)**
High	**35 (65)**	**4 (7)**	**33 (60)**	**5 (10)**	**34 (63)**	**4 (8)**
PR expression	***P* = 0.017**				***P* = 0.0005**	
Neg/Low	**60 (111)**	**26 (47)**	**65 (119)**	**22 (40)**	**55 (101)**	**31 (56)**
High	**13 (24)**	**1 (2)**	**12 (22)**	**2 (3)**	**14 (25)**	**1 (1)**
HER-2 expression						
Neg/Low	58 (105)	21 (38)	58 (106)	19 (34)	53 (96)	24 (44)
High	15 (28)	6 (11)	18 (33)	5 (9)	16 (28)	7 (13)
EGFR expression	***P* = 0.016**		***P* = 0.007**		***P* < 0.0001**	
Neg/Low	**63 (114)**	**19 (34)**	**65 (119)**	**15 (28)**	**61 (110)**	**19 (35)**
High	**10 (18)**	**8 (15)**	**11 (20)**	**8 (15)**	**7 (13)**	**12 (22)**
Ki-67 expression	***P* = 0.027**		***P* = 0.007**		***P* = 0.001**	
Neg/Low	**48 (87)**	**13 (23)**	**50 (92)**	**10 (18)**	**47 (85)**	**13 (24)**
High	**25 (46)**	**14 (26)**	**26 (48)**	**14 (25)**	**22 (40)**	**18 (33)**
p53 expression	***P* = 0.024**		***P* = 0.05**		***P* = 0.013**	
Neg/Low	**57 (103)**	**16 (29)**	**59 (107)**	**14 (26)**	**53 (96)**	**18 (33)**
High	**17 (30)**	**11 (20)**	**18 (33)**	**9 (17)**	**16 (29)**	**13 (24)**

Fisher's exact test and chi-square test.

**Table 7:** Histoclinical correlations of breast cancers according to galectins expression in cancer-associated stroma

Characteristics	Gal-1 / Gal-3 / Gal-9 stromal expression		*P*
	Negative *n* = 104	Triple positive *n* = 22	
Age, yr			0.562
≤ 45	18% (22)	2% (3)	
> 45	65% (81)	15% (19)	
Tumor volume			0.466
≤ 10 cm^3^	48% (55)	9% (10)	
> 10 cm^3^	34% (39)	10% (11)	
Lymph node metastasis			1.000
Negative	52% (63)	12% (14)	
Positive	30% (36)	7% (8)	
SBR-EE grade			**0.003**
I (low)	24% (28)	0% (0)	
III (high)	58% (67)	18% (21)	
Molecular subtype			**< 0.0001**
Triple negative	23% (29)	13% (17)	
HER-2 positive	12% (15)	4% (5)	
Luminal A	42% (53)	0% (0)	
Luminal B (HER-2+)	5% (7)	0% (0)	
ER expression			**< 0.0001**
Neg/Low	41% (51)	17% (22)	
High	42% (53)	0% (0)	
PR expression			**0.023**
Neg/Low	66% (83)	17% (22)	
High	17% (21)	0% (0)	
HER-2 expression			1.000
Neg/Low	64% (80)	14% (17)	
High	18% (22)	4% (5)	
EGFR expression			**0.0003**
Neg/Low	72% (89)	9% (11)	
High	10% (13)	9% (11)	
Ki-67 expression			**0.0005**
Neg/Low	57% (71)	5% (6)	
High	26% (32)	13% (16)	
p53 expression			**0.003**
Neg/Low	65% (81)	8% (10)	
High	18% (22)	10% (12)	

Fisher's exact test and chi-square test.

## DISCUSSION

Triple-negative breast cancer is among the most aggressive breast cancer subtypes. To date, there is no clinically available targeted therapy for patients diagnosed with TNBC and approximately 30% of TNBC patients eventually experience distant relapse. The heterogeneity of TNBC makes predicting treatment difficult and remains a major obstacle for the development of TNBC-specific therapeutic targets. In this study, we report that specific galectin expression signatures at the mRNA and protein levels contribute to the phenotypic heterogeneity of TNBC and segregate subsets of aggressive breast cancer into clinically meaningful subtypes.

Gene or protein expression signatures of cancer tissues are generally obtained from whole tumor homogenates, thus reflecting the expression from all cell types present in the tumor. Given the critical role stromal cells in cancer progression, several groups have thus looked at defining signatures that reflects expression profiles of for both cancer and stromal cells [23]. Such strategy has shown, for instance, that the molecular signature of an immune response is an important prognostic marker in breast cancer and in other cancer forms [23, 24]. Overall, we found that breast cancer stroma was rarely positive for gal-2, -4, -7, and 8. In contrast, gal-1, -3, and -9-positive stroma were frequent, most notably in aggressive molecular subtypes. Interestingly, when released in the extracellular space, these galectins have been shown to contribute induce immune tolerance in various physiological and pathological processes. Such a role as alarmins for galectins has been well documented in pre-eclempsia for several members for the galectin family, including gal-1, -3, and -9 which have been shown to be up-regulated preeclamptic placentas [11, 25–28]. Galectins are also well known for key role in modulating local and systemic anti-tumor responses in cancer [29]. This has been particularly well described for gal-1, induces apoptosis of IFN-γ–producing cells and skews the tumor microenvironment toward a Th2 cytokine profile [30–33]. Gal-1 also contributes to the infiltration of IL-10-producing Treg1 cells to promote the tumor evasion [34]. In fact, we found that in some patients (*n* = 22), all three galectins are expressed in the stroma. Such triple-positive signature was exclusively found in high grade BC and in TNBC (77%) or HER2 (23%) molecular subtypes. Not surprisingly, 75% (16/22) were expressing high levels of Ki-67-positive cells. These results suggest that stromal expression of gal-1, -3, -9 is associated with the most aggressive forms of breast cancer. This possibly explains why absence of gal-3 in preclinical mouse models of breast cancer does not alter tumor progression [35]. Future analyses with a higher number of patients will be needed to determine whether TNBC/HER2 patients that do express all three galectins in their stroma have a worst prognosis as patients than patients who do not express any or less than three of these galectins. It will also be interesting to identify stromal cells that express galectins and whether they do contribute to the presence of galectins in the extracellular space. Although our IHC staining does not allow to determine which galectins are released in the extracellular space and which cells are responsible for this secretion, historically, the presence of extracellular galectins has been attributed to cancer cells. We cannot exclude the possibility, however, that normal and/or cancer-associated stromal cells release soluble galectins. The presence of circulating levels of galectins in normal individuals certainly support sthis possibility. However, given the increasing evidence that intracellular galectins have many cellular functions and the strong cytosolic and nuclear staining that we observed in both cancer and stromal cells, we need to pay a particular attention to their role inside the cells. The emerging evidence that galectins have critical CRD-independent and intracellular functions certainly calls for a refocusing of our efforts on the development of new galectin-specific antagonists.

**Figure 11:**
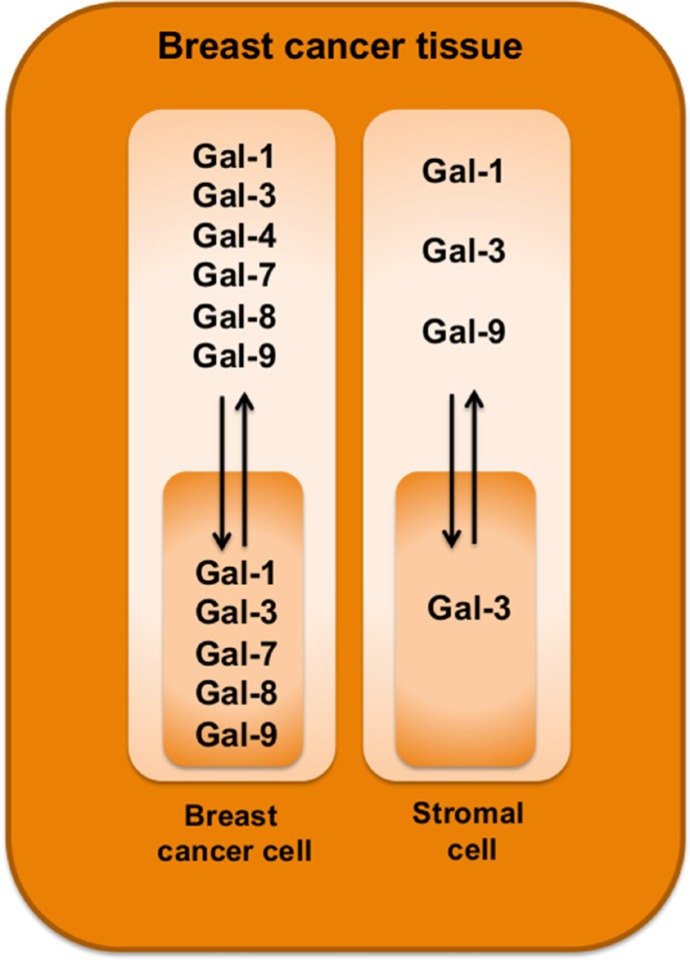
Schematic diagram highlighting galectin expression patterns in breast cancer tissues

It is now well established that it is important to distinguish between stromal and tumor cell signatures to help in defining the heterogeneity of TNBCs and to identify new predictive tools and therapeutic targets. In our study, we have pushed this reasoning one step further by examining the subcellular compartmentalization of a galectins. Our approach was motivated by previous reports showing that members of the galectin family are well known for their heterogeneous pattern of expression and their wide range of biological functions, most notably as modulator of the immune response [29]. Overall, we found that galectins can be potential biomarkers of good and bad prognosis. Gal-1, -3, and -9 expressions in stroma or in tumor cells were all associated with a bad prognosis. Expression of gal-7 was also associated with a bad prognosis, as we previously reported [20]. In contrast to gal-1, -3, and -9, gal-7-positive staining was strictly found in the cytosol and nucleus of epithelial cells of approximately 25% of TNBC and HER2 molecular subtypes. It was not found in luminal A or B subtypes. In the case of gal-2, positive staining was found in all molecular subtypes but its membrane-associated form was also associated with a bad prognosis, as shown by its preferential association with TNBCs and Ki-67-positive staining. Gal-8 was the only galectin that we found was associated with a good prognosis. This association was observed when we took into consideration its nuclear localization in epithelial cells, which correlated with negative/low Ki-67 staining. Overall, these signatures are clearly different from that recently reported in the case of prostate cancer [36]. The authors found that gal-1 was the most abundantly expressed galectin in prostate cancer tissue. In contrast, other galectins (including gal-3, -4, -9, and 12) were expressed at lower levels whereas expression of gal-8 remained unchanged. Although the authors have not examined whether changes in subcellular localization or in the stromal cells occurred, these differences suggest that galectin signatures in cancer are tissue-specific and contributes to the heterogeneity of cancer (Figure 11).

Our results showing that nuclear gal-8 is associated with a good prognosis and that nuclear gal-1 is associated with a negative prognosis are eloquent examples of the importance of taking into account the subcellular localization of proteins with a wide range of subcellular localization. Interestingly, in patients that expressed both nuclear gal-1 and gal-8, the phenotype of gal-8 was clearly dominant. Despite having nuclear gal-1, the 5-year survival rate of patients expressing nuclear gal-8 was 100%. Such dominance for gal-8 also suggests that both galectins have distinct (and contradictory) nuclear functions and that nuclear galectins undergo profound changes during cancer progression. These results uncover the clinical significance of nuclear gal-8 suspected from previous observations in normal and breast tissues [37]. Historically, gal-8 has been mostly recognized “matricellular extracellular protein” that mediates cell-matrix adhesion following binding to cell-surface integrins [38]. Although our approach is not sensitive enough to confirm the presence of gal-8 outside the cell surface, our data showing strong nuclear and cytosolic gal-8 is consistent with other reports that have shown that gal-8 shuttles between the nucleus and the cytosol in cancer cells [39]. Although the molecular mechanism regulating gal-8 (and gal-1) trafficking in the nucleus is currently unknown, it will be interesting to test whether karyopherins are involved. These proteins have been shown to regulate nucleus-cytoplasm transport of galectin-3 [40]. Gal-8, however, is possibly not the only members of the galectin family to be associated with a good prognosis in breast cancer. Preliminary *in silico* analysis using the bc-GenExMiner database shows that high expression of *lgals12, lgals13*, and *lgals14* correlates with a good prognosis in LN-negative and luminal B patients (Supplementary Figure S5). Future work will be needed, however, to determine their expression patterns and the good prognostic potential at the protein level.

Our study is the first study that provides a detailed analysis of the galectin protein signature in molecular subtypes of breast cancer. This signature is clearly different from the mRNA signatures obtained from *in silico* analyses of public databases [41]. For example, our analysis using the bc-GenExMiner database shows that gal-1, -2, -3, -4, -7, -8, and -9 were all expressed at the mRNA level in breast tumor tissues and had a relatively similar distribution among the molecular subtypes. While such databases clearly helps in our understanding of breast cancer and facilitate the identification of novel intrinsic subtypes, caution should be exercised when evaluating the prognostic or therapeutic potential of a given gene, especially genes encoding multifunctional proteins like galectins. A case in point is the relevance of measuring cytoplasmic versus nuclear gal-8 or gal-1 staining. This is a critical issue given that a considerable amount of efforts are underway for the development of galectins inhibitors for the treatment of cancer.

## MATERIALS AND METHODS

### *In silico* analysis

The prognostic module of bc-GenExMiner v3.1 (Breast Cancer Gene-Expression Miner v3.1) [42] was used to correlate survival with each member of galectin family. Gene expression maps represent the percentage of patients with low, intermediate and high gene expression according to molecular subtypes. Kaplan-Meier survival curves (disease free, distance disease free and overall survival) of 4738 patients were obtained from the algorithm BreastMark [14] and the classifier PAM50 [15].

### Patients and tumor materials

A cohort of 213 patients diagnosed with primary breast cancer between 2003 and 2008 at the Centre Hospitalier de l'Université de Montréal (CHUM) was used for the study. Tumors were selected on the basis of the histological diagnosis according to the classification of the Modified Scarff-Bloom-Richardson-Elston-Ellis grading system (SBR-EE) [43]. The cohort consisted of both low-grade and high-grade ductal carcinomas and of carcinomas with medullary features. Estrogen receptor status was positive in all low-grade carcinomas. This study was approved by the research ethics committee (CÉR) of the research centre at the CHUM (study SL05.019), in accordance with the Tri-Council Policy Statement on Research with Human Subjects. Consents directly from patients were not required in this study as per Ethics Board guidelines.

### Tissue microarrays and immunohistochemistry

Formalin-fixed paraffin-embedded material from each primary tumor sample was used to construct tissue microarrays with an automated arrayer design to construct high-density tissue micro-array blocks (ATA-27 Beecher Instruments, Sun Prairie, WI). To that end, triplicate 1 mm cores from each tumor and control tissues were punched out and arrayed into six recipient blocks. For immunohistochemical analysis, three-micrometer thick sections were prepared from each TMA. Immunostaining reactions for each galectin were carried out using the Discovery XT automated immunostainer (Ventana Medical Systems, Tucson, AZ). Deparaffinized sections were incubated in cell conditioning pH 8, except for anti-galectin-2 (pH 6), for antigen retrieval and then with primary antibodies for 1 to 3 hrs: mouse monoclonal anti-galectin-1 (1:50; Novocastra, Leica Biosystems, Newcastle Upon Tyne, United Kingdom), rabbit polyclonal anti-galectin-2 (1:100; Proteintech Group, Chicago, IL, USA), rabbit monoclonal anti-galectin-3 (1:1000; Abcam, Cambridge, MA, USA), goat polyclonal anti-galectin-4 (1:200; Santa Cruz, Santa Cruz, CA, USA), goat polyclonal anti-galectin-7 (1:1000; R&D Systems, Minneapolis, MN, USA), rabbit polyclonal anti-galectin-8 (1:50; Abcam) and rabbit polyclonal anti-galectin-9 (1:100; Abcam). The slides were counterstained with hematoxylin and bicarbonate. Each section was scanned at a high resolution using the Nanozoomer Digital Pathology (Hamamatsu, Bridgewater, NJ). The validation of antibodies specificity was assessed using a tissue microarray of 21 different human normal tissues. Positive and negative controls were evaluated according to publications. The omission of the primary antibody was also used as a negative control. All antibodies were validated by their respective company and anti-galectin-1, 2, 3, 4 and 7 were used in previous publications [20, 44–49].

### Evaluation of immunohistochemical staining

The percentage of staining was scored from 0 to 4 according to the percentage of positive cells displaying galectins expression within a sample (0 (0–9%); 1 (10–25%); 2 (26–50%); 3 (51–75%); 4 (76–100%). The intensity of staining was also scored from 0 to 4, with a score of 0 representing no staining and a score of 4 representing the strongest staining observed. Histological scores were calculated by adding both scores and a strong expression was defined by a score of 6 to 8. This scoring system is somewhat comparable to the Allred score [48, 49].

### Statistical analysis

Kaplan-Meier curves and relationship between proteins expression were assessed using GraphPad Prism 5.00 (GraphPad Software, San Diego, CA). For Fisher's exact test, chi-square test and spearman analysis, SPSS Statistics (IBM Corporation, Armoncon, NY) was used. A *P* value of 0.05 or less was considered statistically significant.

## SUPPLEMENTARY MATERIALS FIGURES AND TABLES


