# Supplementary material for: Galectin signatuares contribute to the heterogeneity of breast cancer and provide new prognostic information and therapeutic targets
Source: Oncotarget. 2016 Feb 27;7(14):18183–203. doi: 10.18632/oncotarget.7784 (PMC4951281; doi:10.18632/oncotarget.7784)
Supplement: Supplementary file 1 [file oncotarget-07-18183-s001.pdf]

Galectin signatures contribute to the heterogeneity of breast cancer and provide new prognostic information and therapeutic targets

Supplementary Materials

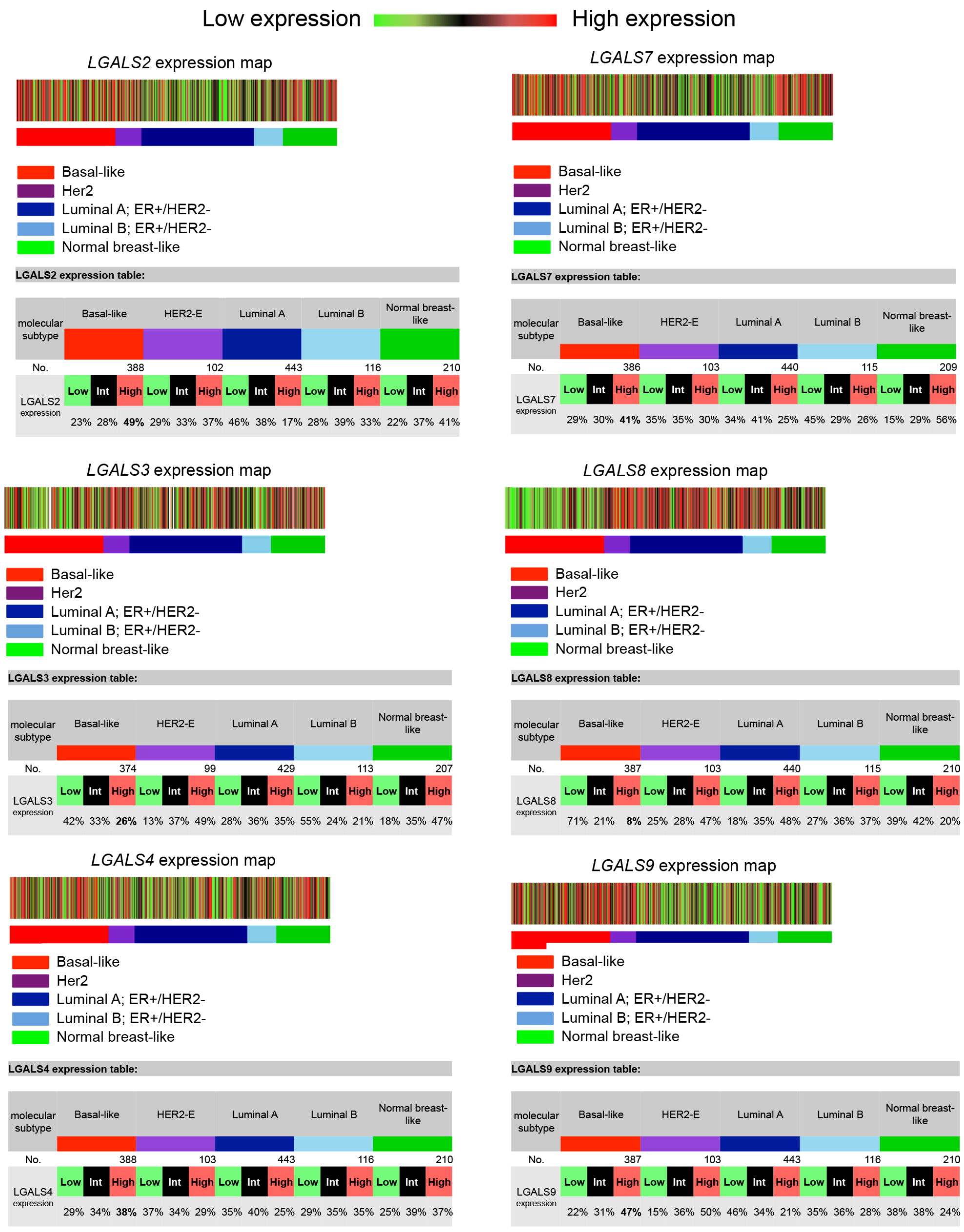

Supplementary Figure S1: *In silico* analysis of galectins mRNA expression of human breast cancer (bc-GenExMiner). Each map shows the percentage of patients with low, medium and high expression of *lgals2, 3, 4, 7, 8* and *9* mRNA in each molecular subtype: Basal-like (ER-, HER-2-), HER-2E (HER-2 enriched), Luminal A (ER+, HER-2-, low proliferation) and Luminal B (ER+, HER-2-, high proliferation).

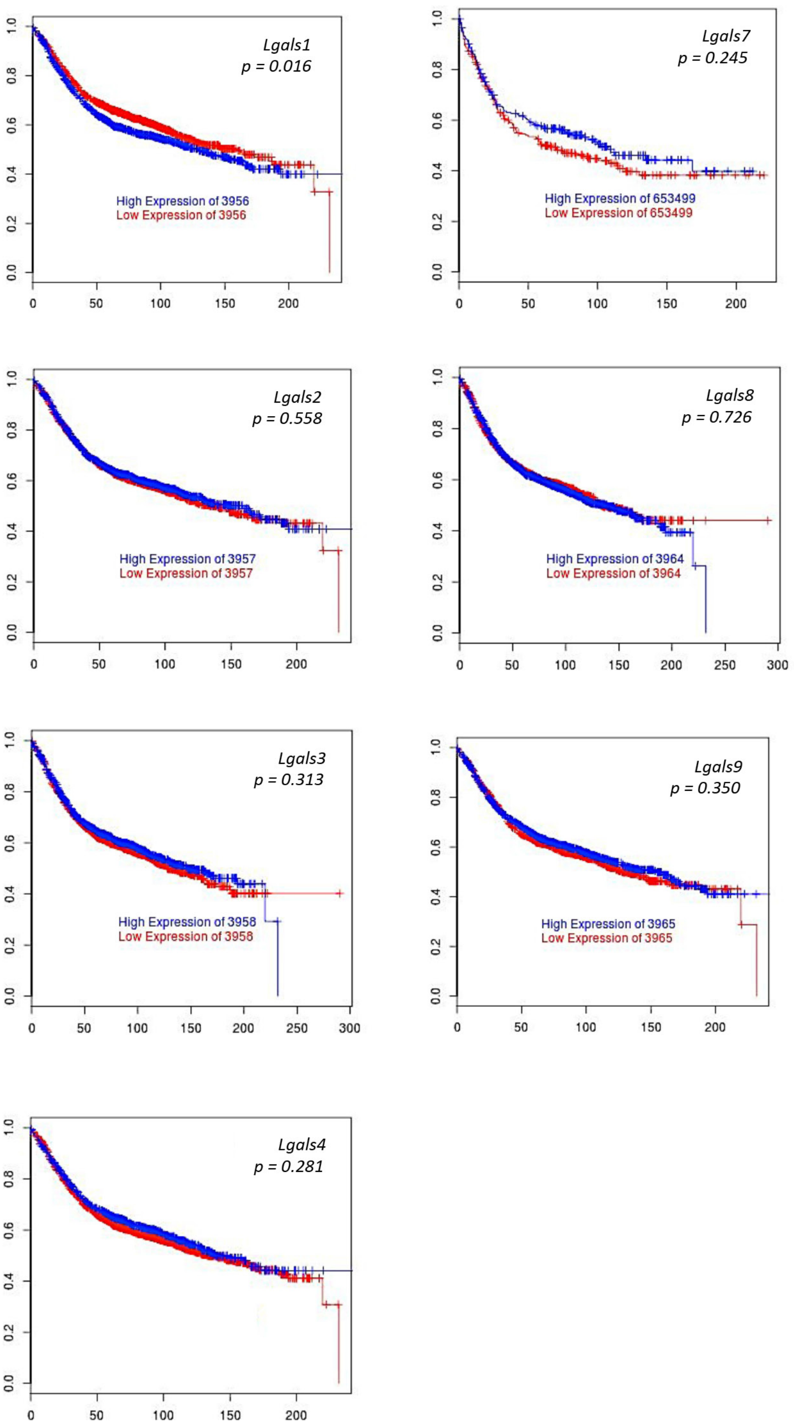

Supplementary Figure S2: Effect of *galectin* gene expression on disease-free survival (DFS) of patients with breast cancer, independently of the LN status and across the molecular subtypes. Kaplan-Meier estimates of overall survival in breast cancer patients expressing low or high *galectin* expression. These figures were generated using BreastMark public database.

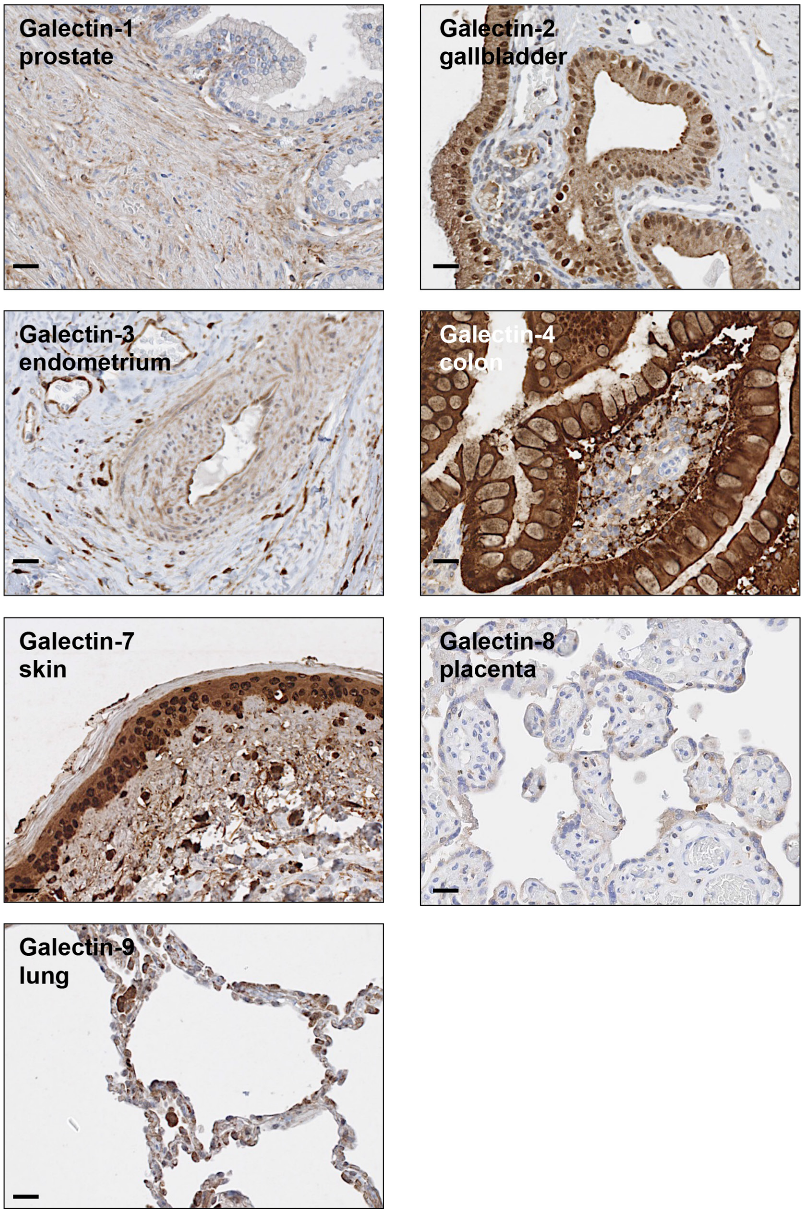

Supplementary Figure S3: Representative IHC staining showing positive controls of galectins expression in normal tissues.

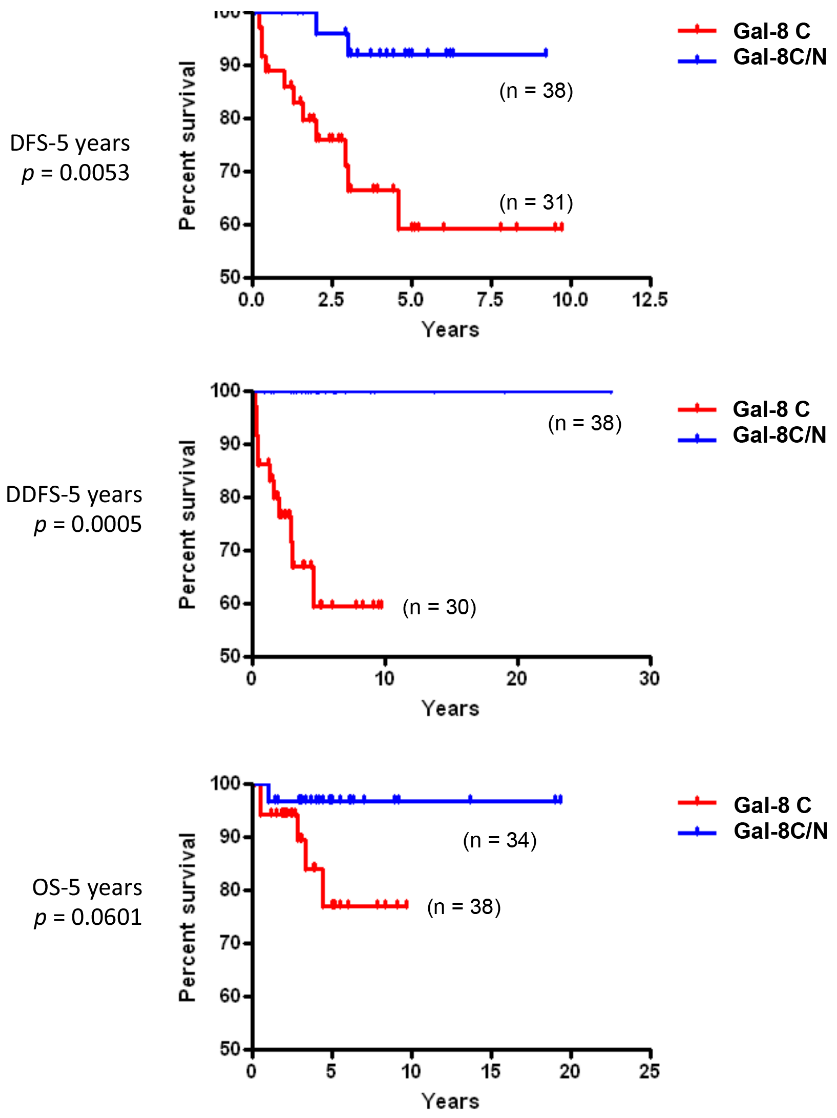

Supplementary Figure S4: Prognostic potential of galectin in across molecular subtypes of breast cancer. Kaplan-Meier estimates of 5-year DFS in breast cancer patients with low or high galectin expression independently of the molecular subtype.

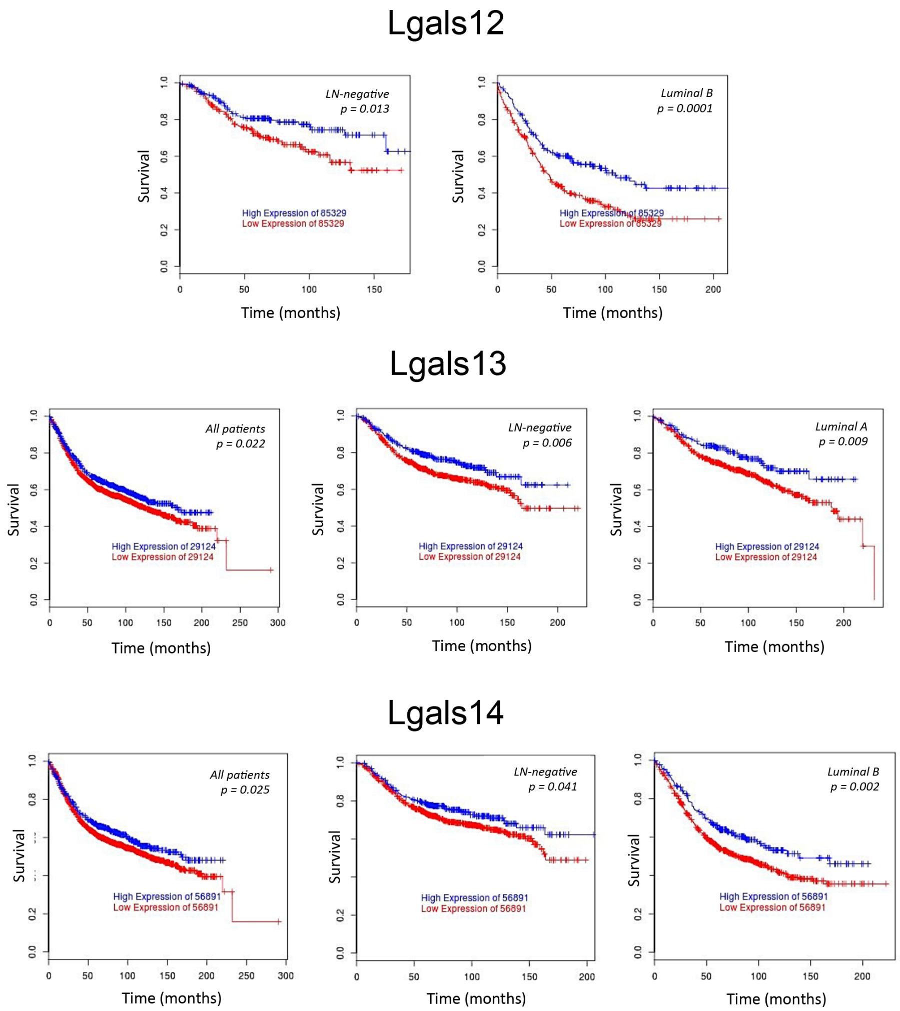

Supplementary Figure S5: Prognostic potential of *galectin-12, -13* and *-14* genes in breast cancer. Kaplan-Meier estimates of overall survival in breast cancer patients expressing low or high *galectin* expression. These figures were generated using BreastMark public database.

Supplementary Table S1: Antibodies validation

	Company	Validation	Reference
Gal-1	Novocastra	Prostate	Ellerhorst *et al*, 1999
Gal-2	Proteintech Group	Gallbladder	Saal *et al*, 2005
Gal-3	Abcam	Endometrium	Brustmann *et al*, 2003
Gal-4	Santa Cruz	Colon	Huflejt *et al*, 2004
Gal-7	R & D Systems	Skin	Magnaldo *et al*, 1998
Gal-8	Abcam	Placenta	Kolundzic *et al*, 2011
Gal-9	Abcam	Lung	Matsumoto *et al*, 2013

Supplementary Table S2: Histoclinical correlations of triple negative breast cancers according to galectins expression in cancer cells

	Gal-1 %(n)		Gal-2 %(n)		Gal-3 %(n)		Gal-4 %(n)		Gal-7 %(n)		Gal-8 %(n)		Gal-9 %(n)	
	Neg/ Low	High	Neg/ Low	High	Neg/ Low	High	Neg/ Low	High	Neg/ Low	High	Neg/ Low	High	Neg/ Low	High
Age, yr							***P* = 0.013**							
≤ 45	7 (5)	15 (11)	4 (3)	19 (14)	3 (2)	19 (14)	**16 (12)**	**7 (5)**	18 (13)	6 (4)	4 (3)	18 (13)	13 (9)	11 (8)
> 45	29 (21)	49 (36)	12 (9)	65 (48)	19 (14)	58 (42)	**73 (54)**	**4 (3)**	57 (41)	19 (14)	25 (18)	53 (39)	42 (30)	35 (25)
Tumor volume														
≤ 10 cm^3	18(12)	30(20)	7(5)	38(26)	10 (7)	35 (24)	38 (26)	7 (5)	36 (24)	10 (7)	13 (9)	34 (23)	24 (16)	22 (15)
> 10 cm^3	18(12)	34(23)	9(6)	46(31)	13 (9)	41 (28)	50 (34)	4 (3)	42 (28)	12 (8)	16 (11)	37 (25)	30 (20)	24 (16)
Lymph node metastasis														
Negative	29 (21)	45 (33)	8 (6)	65 (48)	17 (12)	54 (39)	66 (49)	5 (4)	56 (40)	17 (12)	18 (13)	53 (39)	36 (26)	35 (25)
Positive	7 (5)	19 (14)	8 (6)	19 (14)	6 (4)	24 (17)	23 (17)	5 (4)	19 (14)	8 (6)	11 (8)	18 (13)	18 (13)	11 (8)
Recurrence	***P* = 0.021**				***P* = 0.019**									
No	**30 (21)**	**32 (23)**	7 (5)	53 (38)	**19 (13)**	**41 (29)**	56 (40)	4 (3)	46 (32)	14 (10)	14 (10)	45 (32)	34 (24)	26 (18)
Yes	**7 (5)**	**31 (22)**	8 (6)	32 (23)	**3 (2)**	**37 (26)**	35 (25)	6 (4)	30 (21)	10 (7)	16 (11)	25 (18)	19 (13)	21 (15)
Distant metastasis														
No	31 (22)	48 (34)	10 (7)	67 (48)	20 (14)	56 (39)	69 (50)	7 (5)	57 (40)	19 (13)	24 (17)	52 (37)	40 (28)	36 (25)
Yes	6 (4)	16 (11)	6 (4)	18 (13)	1 (1)	23 (16)	21 (15)	3 (2)	19 (13)	6 (4)	6 (4)	18 (13)	13 (9)	11 (8)
Death			***P* = 0.015**											
No	34 (24)	51 (36)	**8 (6)**	**75 (54)**	19 (13)	64 (45)	74 (53)	10 (7)	63 (44)	20 (14)	24 (17)	59 (42)	43 (30)	40 (28)
Yes	3 (2)	13 (9)	**7 (5)**	**10 (7)**	3 (2)	14 (10)	17 (12)	0 (0)	13 (9)	4 (3)	6 (4)	11 (8)	10 (7)	7 (5)
Ki-67											***P* = 0.009**			
Neg/ Low	8 (6)	21 (15)	5 (4)	23 (17)	6 (4)	24 (17)	24 (18)	4 (3)	21 (15)	8 (6)	**15 (11)**	**14 (10)**	21 (15)	8 (6)
High	27 (20)	44 (32)	11 (8)	61 (45)	17 (12)	54 (39)	65 (48)	7 (5)	54 (39)	17 (12)	**14 (10)**	**58 (42)**	33 (24)	38 (27)

Fisher's exact test and chi-square test.
